# Phytochemical Investigation of *Aquilaria agallocha* and Identification of a Diarylheptanoid Exhibiting Anti-Tau Aggregation Activity

**DOI:** 10.3390/biomedicines13122855

**Published:** 2025-11-23

**Authors:** Yeo Rang Cho, Jiyeon Kim, Bora Kim, Dong Min Kang, Yun Kyung Kim, Jin-Chul Kim, Sungsu Lim, Ki Hyun Kim

**Affiliations:** 1 School of Pharmacy, Sungkyunkwan University, Suwon 16419, Republic of Korea; yr6755@g.skku.edu (Y.R.C.); delucete@naver.com (J.K.); wvely_x@naver.com (B.K.); 2 Department of Life Sciences, Korea University, Seoul 02841, Republic of Korea; swize3657@kist.re.kr; 3 Brain Science Institute, Korea Institute of Science and Technology (KIST), Seoul 02792, Republic of Korea; yunkyungkim@kist.re.kr; 4 Division of Bio-Medical Science & Technology, KIST School, University of Science and Technology (UST), Seoul 02792, Republic of Korea; 5 Natural Products Informatics Center, Korea Institute of Science and Technology (KIST), Gangneung 25451, Republic of Korea; jckim@kist.re.kr; 6 Natural Product Applied Science, KIST School, University of Science and Technology (UST), Gangneung 25451, Republic of Korea

**Keywords:** *Aquilaria agallocha*, diarylheptanoid, LC/MS, tau

## Abstract

Agarwood from *Aquilaria agallocha*, known as chim-hyuang in Korea, is widely distributed throughout Southeast Asia and has traditionally been used to treat asthma, pain, and gastrointestinal disorders. As part of our ongoing efforts to identify bioactive metabolites from natural sources, a phytochemical investigation of the EtOAc fraction of *A. agallocha* extract led to the isolation and identification of four compounds, *N*-*trans*-feruloyltyramine (**1**), (3*R*,5*R*)-octahydrocurcumin (**2**), 1,7-bis(4-hydroxyphenyl)heptane (**3**), and *trans*-caffeoyltyramine (**4**), via HPLC purification and LC/MS-based analysis. Structural elucidation of the isolated compounds was achieved using NMR spectroscopy, LC/MS, and high-resolution electrospray ionization mass spectrometry (HR-ESIMS). The absolute configuration of compound **2** was further confirmed by optical rotation and electronic circular dichroism (ECD) analyses. All isolated compounds (**1**–**4**) were evaluated for their inhibitory activity against tau protein aggregation. Notably, compound **2** exhibited a 43.7% reduction in tau aggregation at 20 μM, without cytotoxicity at the same concentration. These findings indicate that phytochemicals from *A. agallocha*, particularly the diarylheptanoid compound **2**, hold promise as natural lead candidates for the development of therapeutic agents targeting tau protein aggregation.

## 1. Introduction

The plant genus *Aquilaria*, belonging to the family Thymelaeaceae, comprises evergreen tropical trees renowned for producing aromatic resins and essential oils. These species are predominantly distributed across Southeast Asia, including Bangladesh, Indonesia, India, and Malaysia [1]. Among them, *Aquilaria agallocha* is particularly valued for its resinous heartwood, which yields a highly fragrant resin known as chen-xiang in China and chim-hyuang in Korea. This resin is formed as a defensive response to mechanical injury or biotic stress, such as fungal infection or insect attack [2]. For centuries, agarwood has been widely used in traditional medicine throughout Asia. In China, powdered agarwood has been prescribed for the treatment of asthma, pain, and gastrointestinal disorders, whereas in Tibetan medicine, it has been applied to manage psychological and emotional conditions [3,4].

In recent decades, pharmacological studies have confirmed that extracts of *A. agallocha* exhibit diverse biological activities, including cytotoxicity against lung cancer cells, hepatoprotective effects, anti-inflammatory activity via suppression of nitric oxide (NO) production, and antioxidant and antibacterial properties against *Bacillus subtilis* [5,6,7]. Phytochemical investigations have led to the identification of a wide range of secondary metabolites from the agarwood of *A. agallocha*, including sesquiterpenes, triterpenes, 2-(2-phenylethyl)chromone derivatives—the major chemical constituents of agarwood—and various phenolic compounds [8]. Several of these abundant metabolites have demonstrated notable bioactivities in experimental models, such as anti-inflammatory effects mediated by NO inhibition and the upregulation of brain-derived neurotrophic factor (BDNF) expression [9,10].

Neurodegenerative disorders such as Alzheimer’s disease and related tauopathies are characterized by abnormal protein aggregation, with tau aggregation recognized as a central pathogenic event [11]. Although synthetic inhibitors of tau aggregation have been explored, their development has been hampered by limitations related to safety, bioavailability, and therapeutic efficacy, thereby increasing interest in natural products as alternative sources of structurally diverse and biologically safe compounds [12,13]. Given that phytochemicals from *A. agallocha* have been reported to modulate neuronal pathways, including enhancement of BDNF signaling [9,10] and activation of anti-inflammatory and antioxidant pathways [14,15], this species represents a promising reservoir for the discovery of novel agents targeting tau pathology. Moreover, diarylheptanoids—including octahydrocurcumin analogs—have been reported to modulate oxidative stress [16,17] and endoplasmic reticulum (ER) stress-related pathways in neuronal cells [18], both of which are closely associated with tau oligomerization. These mechanistic properties further support their potential relevance in tau-targeted research.

Despite extensive research on the chemical constituents of *A. agallocha*, most studies to date have focused on well-known and abundant components, particularly sesquiterpenes and 2-(2-phenylethyl)chromone derivatives. In contrast, minor or less-characterized metabolites—such as diarylheptanoids and phenylpropanoid amides—remain largely unexplored, and their potential neuroprotective or tau modulatory effects have not been investigated. This knowledge gap highlights an opportunity to identify new bioactive constituents with therapeutic relevance.

As part of our ongoing efforts to discover new bioactive natural compounds [19,20,21,22,23,24], we conducted a phytochemical investigation of the EtOAc fraction of *A. agallocha*, leading to the isolation of four compounds: *N*-*trans*-feruloyltyramine (**1**), (3*R*,5*R*)-octahydrocurcumin (**2**), 1,7-bis(4-hydroxyphenyl)heptane (**3**), and *trans*-caffeoyltyramine (**4**), through successive purification using HPLC. The structures of compounds **1**–**4** were elucidated by NMR spectroscopy and high-resolution electrospray ionization mass spectrometry (HR-ESIMS), and the absolute configuration of compound **2** was confirmed by specific optical rotation and electronic circular dichroism (ECD) calculations. In this study, we further evaluated these underexplored constituents as potential therapeutic candidates for neurodegenerative disease, with particular emphasis on the tau aggregation inhibitory activity of the diarylheptanoid (*3R,5R*)-octahydrocurcumin.

## 2. Materials and Methods

### 2.1. General Experimental Procedures

The equipment and devices used in the analyzes and experiments are listed in Table 1.

### 2.2. Plant Material

**Figure 1:**
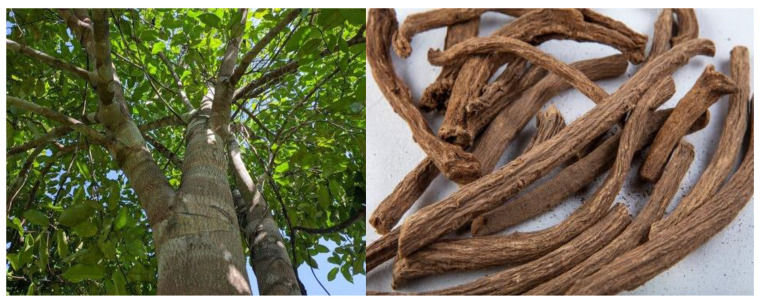
Photographs of *A. agallocha* agarwood.

The resinous wood of *A. agallocha* was collected in Indonesia in June 2022. The plant material was authenticated by Prof. K. H. Kim, one of the authors of this study. A voucher specimen (CH-2022) has been deposited in the herbarium of the School of Pharmacy, Sungkyunkwan University, Suwon, Republic of Korea (Figure 1).

### 2.3. Extraction and Isolation

The powdered resinous wood of *A. agallocha* (70.0 g) was extracted with 700 mL of 80% methanol at room temperature for 12 h. This procedure was repeated three times. The combined extracts were filtered and concentrated under reduced pressure to yield 24.7 g of crude extract. The crude extract was suspended in 700 mL of distilled water and successively partitioned three times with *n*-hexane, dichloromethane (CH_2_Cl_2_), ethyl acetate (EtOAc), and *n*-butanol (*n*-BuOH), affording 144.0 mg, 1.2 g, 12.5 g, and 10.1 g of the respective fractions. The EtOAc fraction (12.5 g) was subjected to normal-phase silica gel open-column chromatography and eluted using a gradient of CH_2_Cl_2_/MeOH (50:1 → 30:1 → 10:1 → 1:1 → 100% MeOH), yielding four fractions (CE1–CE4). Fraction CE2 (758 mg) was further separated by preparative HPLC on a Hector C18 column (250 × 21.2 mm, 5 μm; flow rate: 5 mL/min) with a gradient of 73–100% aqueous MeOH, giving six subfractions (CE21–CE26). Subfraction CE21 (119 mg) was purified by preparative HPLC on a Hector C18 column under the same gradient conditions to afford five subfractions (CE211–CE215). Subfraction CE212 (80 mg) was purified by semi-preparative HPLC on a Phenomenex Luna C18 column (250 × 10 mm, 10 μm; flow rate: 2 mL/min) using isocratic 50% MeOH to yield compounds **1** (6.9 mg, *t_R_* 24 min) and **2** (16.9 mg, *t_R_* 33 min). Subfraction CE25 (147 mg) was chromatographed on a Sephadex LH-20 column eluted with 80% MeOH to provide seven subfractions (CE251–CE257). Subfraction CE256 (20 mg) was purified by semi-preparative HPLC on a Phenomenex Luna C18 column using 75% aqueous MeOH to yield compound **3** (4.2 mg, *t_R_* 41 min). Fraction CE3 (350 mg) was subjected to preparative HPLC on a Hector C18 column using a gradient of 55–80% aqueous MeOH to obtain four subfractions (CE31–CE34). Subfraction CE31 (56 mg) was further purified by semi-preparative HPLC on a Phenomenex Luna C18 column with a gradient of 35–60% aqueous MeOH to afford compound **4** (4.0 mg, *t_R_* 30 min).

### 2.4. UHPLC–QTOF–MS/MS Analysis and GNPS Molecular Networking

Four solvent-partitioned fractions of the *A. agallocha* extract were subjected to MS/MS analysis using an Agilent 1290 Infinity II UHPLC system coupled to a G6545B quadrupole time-of-flight (Q-TOF) mass spectrometer (Agilent Technologies, Santa Clara, CA, USA) equipped with an electrospray ionization (ESI) source. Each sample was dissolved in HPLC-grade methanol (1 mg/mL), filtered, and 200 μL of each solution was transferred to an HPLC autosampler vial. Chromatographic separation was carried out on an Acquity^®^ UPLC BEH C18 column (150 mm × 2.1 mm, 1.7 μm). The gradient system was optimized according to the polarity of each fraction. For the *n*-hexane and dichloromethane fractions, the mobile phase gradient was programmed as follows: 50–100% MeCN (0–14 min), 100% MeCN (14–17 min), 100–50% MeCN (17–17.1 min), and 50% MeCN (17.1–20 min). For the EtOAc and *n*-BuOH fractions, the following gradient was applied: 10–100% MeCN (0–14 min), 100% MeCN (14–17 min), 100–10% MeCN (17–17.1 min), and 10% MeCN (17.1–20 min). The flow rate was 0.3 mL/min, and the injection volume was 5 μL. MS/MS data were acquired in positive ion mode under the following ESI conditions: sheath gas temperature, 350 °C; sheath gas flow, 11 L/min; drying gas temperature, 320 °C; drying gas flow, 8 L/min; nebulizer pressure, 35 psig; nozzle voltage, 1000 V; fragmentor voltage, 175 V; skimmer voltage, 65 V; octopole RF, 750 V. The mass range was set to *m*/*z* 50–1200, with fixed collision energies of 10, 20, and 40 V, a maximum of five precursors per cycle, and the isotope model set to “common organic molecules.” Raw data files (.d format) were converted to .mzML using MSConvert (version 3.0), and the resulting .mzML files were uploaded to the GNPS platform via WinSCP for molecular networking analysis. Molecular networking was performed following the GNPS workflow described previously [25], and the resulting networks were visualized and analyzed using Cytoscape software (version 3.10.3).

### 2.5. 3D Visualization of LC/MS Data

LC/MS analyses were performed on an Agilent 1200 Series HPLC system equipped with a diode array detector and a 6130 Series electrospray ionization (ESI) mass spectrometer. Chromatographic separation was carried out on a Kinetex C18 column (100 × 2.1 mm, 5 μm, 100 Å; Phenomenex, Torrance, CA, USA) at a flow rate of 0.3 mL/min. The mobile phase gradient was programmed from 10% MeOH–H_2_O to 100% MeOH over 52 min as follows: 10–100% MeOH (0–30 min), 100% MeOH (30–41 min), 100–10% MeOH–H_2_O (41–42 min), and 10% MeOH–H_2_O (42–52 min). LC/MS data were processed using MZmine 4.2.0, and three-dimensional chromatographic visualizations were generated. The data processing parameters were as follows: mass range, *m*/*z* 100–1000; retention time resolution, 500; *m*/*z* resolution, 500; MS level, 1; ionization mode, positive; and spectrum type, centroid.

### 2.6. Computational Analysis of Electronic Circular Dichroism (ECD) Spectra

Electronic circular dichroism (ECD) calculations for conformers **2a** and **2b** were performed using time-dependent density functional theory (TD-DFT) at the B3LYP/6-31+G(d,p) level of theory, following previously described literature [26]. The theoretical ECD spectra were generated by summing the individual electronic transitions, each represented by a Gaussian function with a bandwidth (σ) of 0.20 eV at 1/*e* of its maximum intensity [26]. The excitation energies (Δ*E_i_*) and rotatory strengths (*R_i_*) obtained from the TD-DFT calculations were Boltzmann-weighted according to the relative populations of the conformers to produce the final computed spectra. The resulting ECD curves were visualized and plotted using SigmaPlot 14.0 [27].

### 2.7. Tau-BiFC Cell Culture

HEK293 tau–BiFC cells were maintained in Dulbecco’s modified Eagle medium (DMEM) supplemented with 10% fetal bovine serum (FBS), 100 U/mL penicillin, 100 μg/mL streptomycin, and 100 μg/mL Geneticin (G418). Cells were cultured at 37 °C in a humidified incubator containing 5% CO_2_.

### 2.8. Compound Treatment and Image Analysis

HEK293 tau–BiFC cells were seeded into μ-clear 384-well plates. The following day, cells were treated with thapsigargin (TG, 0.05 μM) to induce tau oligomerization, followed by treatment with compounds **1**–**4** at final concentrations of 1.25, 5, and 20 μM. After 48 h, cell nuclei were counterstained with Hoechst (0.3 μg/mL).

Fluorescence images of BiFC (λ_ex_ = 460–490 nm; λ_em_ = 500–550 nm) and Hoechst (λ_ex_ = 355–385 nm; λ_em_ = 430–500 nm) were automatically acquired using an Operetta^®^ CLS high-content imaging system (PerkinElmer, Shelton, CT, USA) equipped with a built-in sCMOS camera and an 8-channel LED light source. All images were collected using the same 20× water immersion objective (30% LED power, 20 ms exposure time) to ensure identical magnification and image settings across all samples. BiFC fluorescence intensity and total nuclei counts were quantified using Harmony 4.9 software (PerkinElmer). Integrated tau–BiFC intensity was calculated as the product of cell area, mean fluorescence intensity, and number of cells. Data are presented as mean ± standard deviation (SD), and graphs were generated using Prism 10 (GraphPad).

## 3. Results and Discussion

### 3.1. LC-MS/MS-Based Molecular Networking Analysis

**Figure 2:**
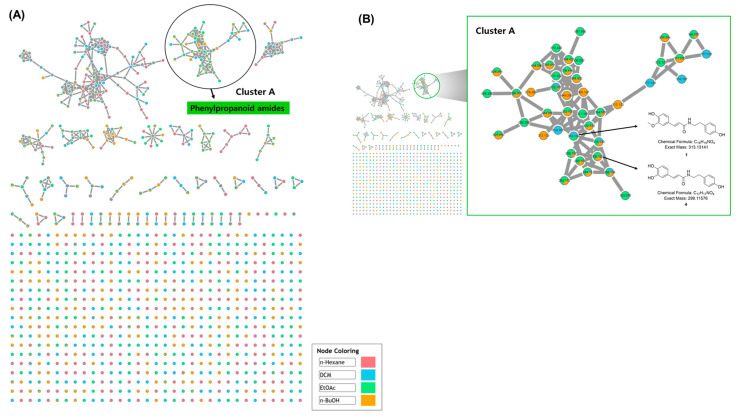
(**A**) GNPS molecular networking analysis of MS/MS data obtained from four solvent-partitioned fractions of the *A. agallocha* extract in positive ion mode. Each node in the network is represented as a pie chart, with pink, blue, green, and orange sectors indicating the relative MS intensities of features detected in the four respective fractions. (**B**) Enlarged view of Cluster A, highlighting nodes annotated as phenylpropanoid amide–type metabolites.

The powdered resinous wood of *A. agallocha* was extracted with 80% methanol and subsequently partitioned into four fractions using *n*-hexane, dichloromethane (DCM), EtOAc, and *n*-BuOH. To investigate the metabolite profile of the *A. agallocha* extract, four solvent-partitioned fractions were analyzed by ultra-high-performance liquid chromatography coupled with quadrupole time-of-flight mass spectrometry (UHPLC–QTOF–MS), and MS/MS spectra were acquired for each fraction. The obtained datasets were subsequently analyzed using the GNPS web platform (https://gnps.ucsd.edu, accessed on 1–5 October 2025). As a result, molecular networking generated a total of 46 clusters, which were automatically grouped based on structural similarities and chemical classes (Figure 2A). Among these, phenylpropanoid amide-type metabolites were identified in Cluster A through GNPS spectral library matching. Within this cluster, the precursor ion at *m*/*z* 314.139 [M + H]^+^ was putatively annotated as feruloyltyramine, and several structurally related nodes within the *m*/*z* 260–320 range were also detected (Figure 2B). GNPS analysis further revealed that phenylpropanoid amide derivatives were predominantly present in the EtOAc fraction, with corresponding nodes clearly represented in Cluster A. Several studies have reported that phenylpropanoid amides exhibit diverse pharmacological activities, including anti-inflammatory, antioxidant, antimicrobial, and anticancer effects [28]. In particular, clovamide isolated from *Theobroma cacao* L. has shown strong anti-inflammatory activity by suppressing the production of superoxide anions and reducing the synthesis of TNF-α and IL-6 in human monocytes [29]. Another group of phenylpropanoid amides, the avenanthramides derived from *Avena sativa* (oat), demonstrate potent anti-inflammatory and antioxidant effects through free radical scavenging activity and inhibition of NF-κB signaling [30]. Although phenylpropanoid amides are valuable natural compounds with diverse biological activities, studies on their occurrence and bioactivities in *A. agallocha* remain limited. Based on these findings, the EtOAc fraction was selected for subsequent isolation of secondary metabolites, particularly those that have been rarely characterized in *A. agallocha*.

### 3.2. Isolation and Structural Elucidation of Compounds **1**–**4** from the EtOAc Fraction

**Figure 3:**
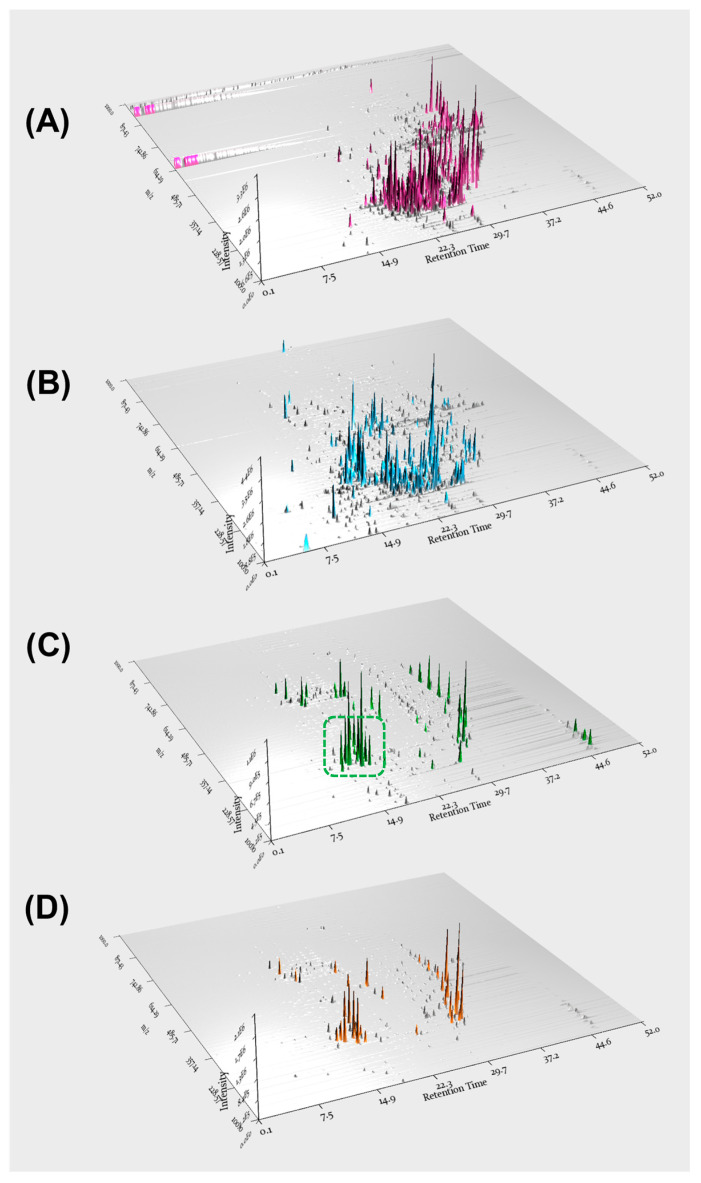
3D plot visualizations of the soluble fractions obtained from the *A. agallocha* extract: (**A**) *n*-hexane, (**B**) CH_2_Cl_2_, (**C**) EtOAc, and (**D**) *n*-BuOH. The green dotted box in panel (**C**) highlights the region corresponding to phenylpropanoid amides, showing a characteristic *m*/*z* range of approximately 290–400.

**Figure 4:**
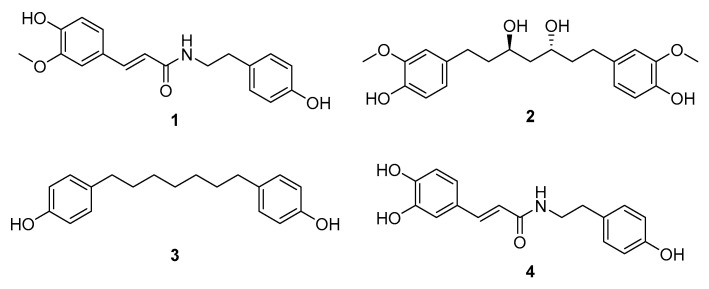
Chemical structures of compounds **1**–**4**.

Based on molecular networking analysis as well as LC/MS analysis, the EtOAc fraction was found to contain key constituents of *A. agallocha*, including phenylpropanoid amides and diarylheptanoids. Furthermore, LC/MS data from the four fractions were processed using MZmine 4.2.0 in positive ion mode, and 3D plot visualizations were generated (Figure 3). These plots demonstrated the presence of diverse secondary metabolites in each fraction and confirmed that the EtOAc fraction was enriched in phenylpropanoid amide–type metabolites, characterized by a distinct *m*/*z* range of 280–320. In addition, features corresponding to diarylheptanoids were observed as major constituents of this fraction, appearing predominantly within the *m*/*z* 290–400 range. The EtOAc fraction was therefore subjected to repeated chromatographic separation, including open-column chromatography, preparative HPLC, and semi-preparative HPLC, guided by LC/MS profiling to isolate phenylpropanoid amides and diarylheptanoids. As a result, four compounds were successfully isolated; two phenylpropanoid amides (**1** and **4**) and two diarylheptanoids (**2** and **3**). Based on comparison of their NMR spectroscopic data with previously reported values, the structures of the isolated compounds were identified as *N*-*trans*-feruloyltyramine (**1**) [31], (3*R*,5*R*)-octahydrocurcumin (**2**) [32], 1,7-bis(4-hydroxyphenyl)heptane (**3**) [33], and *trans*-caffeoyltyramine (**4**) [34] (Figure 4).

**Figure 5:**
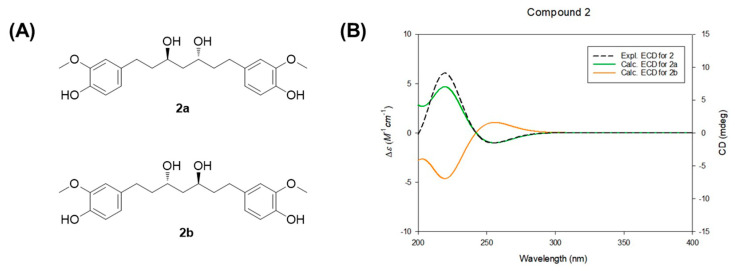
(**A**) Two enantiomers of compound **2** for ECD calculations. (**B**) Experimental and calculated ECD spectra of compound **2**.

The determination of absolute configuration in diarylheptanoids with some hydroxy groups is often challenging. In this work, the absolute configuration of compound **2** was confirmed through comparison of its NMR data and optical rotation with previously reported values. In earlier ^1^H NMR data for *meso*-type octahydrocurcumin, the methylene protons at C-4 appeared as distinct triplets at *δ*_H_ 1.52 (1H, t, *J* = 14.0 Hz) and 1.62 (1H, t, *J* = 14.0 Hz) [35]. However, in (3*R*,5*R*)-octahydrocurcumin and (3*S*,5*S*)-octahydrocurcumin, these protons were observed as triplets at *δ*_H_ 1.54 (2H, t-like) and 1.58 (2H, t), respectively [32,35]. In the spectrum of compound **2**, the C-4 methylene protons were also detected at *δ*_H_ 1.55 (2H, *m*), indicating that compound **2** possesses a configuration different from that of *meso*-octahydrocurcumin. In addition to the ^1^H NMR evidence, the specific rotation value of compound **2**, ${\left[\alpha \right]}_{D}^{20}$ +8.4 (*c* 0.08, MeOH), further supported its assignment as (3*R*,5*R*)-octahydrocurcumin. This is consistent with previous findings that the (3*S*,5*S*)-isomer exhibits an opposite sign of optical rotation [35]. To more rigorously establish the absolute configuration, electronic circular dichroism (ECD) calculations were performed for the two enantiomers (**2a** and **2b**). The experimental ECD spectrum of compound **2** showed strong agreement with the calculated spectrum of isomer **2a** (3*R*,5*R*), corroborating its absolute configuration and aligning with previously reported data [36] (Figure 5).

### 3.3. Inhibition of TG-Induced Tau Oligomerization by Compound **2** in Tau-BiFC Cells

**Figure 6:**
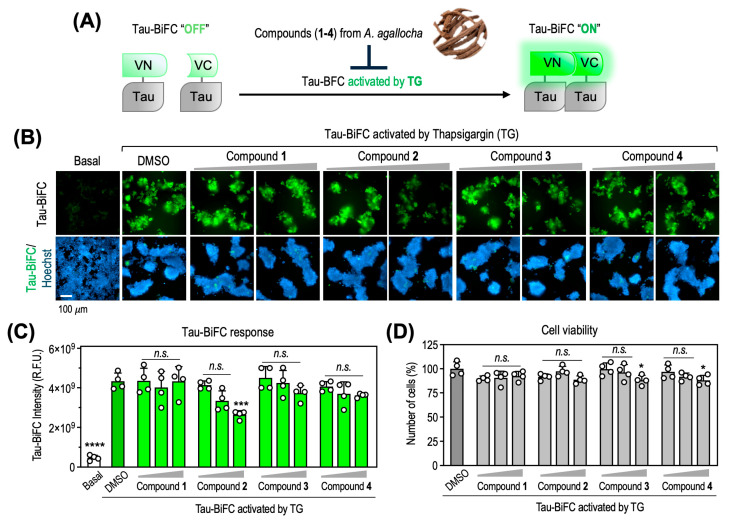
Effects of compound **2** on TG-induced tau oligomerization and cell viability in tau-BiFC cells. (**A**) Schematic diagram of the tau-BiFC system. Full-length tau is fused to split Venus fragments (VN173 and VC155), which reconstitute fluorescence upon tau oligomerization under stress conditions. For the tau-BiFC assay, cells were treated with each compound in the presence of TG. (**B**) Representative BiFC fluorescence images of tau-BiFC cells treated with compounds **1**–**4** (1.25, 5 and 20 μM) in the presence of TG (0.05 μM). Nuclei were counterstained with Hoechst (blue). Scale bar, 100 μm. (**C**) Quantification of tau-BiFC fluorescence intensity. (**D**) Cell viability determined by Hoechst-stained nuclei counting. Data were analyzed by one-way ANOVA followed by Dunnett’s multiple comparisons test; * *p <* 0.05, *** *p <* 0.001, **** *p <* 0.0001 vs. TG-treated control (DMSO).

To evaluate the biological effects of the isolated compounds on tau pathology, we employed a tau–BiFC cellular model [37]. This system enables real-time monitoring of tau oligomerization based on reconstitution of a split fluorescent protein upon tau–tau interactions. Tau–BiFC cells were treated with increasing concentrations of each compound (**1**–**4**; 1.25, 5, and 20 μM) in the presence of thapsigargin (TG), an inducer of endoplasmic reticulum stress [38]. TG markedly enhanced tau oligomerization, inducing a 9.9 ± 2.9-fold increase in BiFC fluorescence relative to basal levels at 48 h (Figure 6A,B). Among the tested compounds, compound **2** significantly reduced TG-induced tau oligomerization at 20 μM, resulting in a 43.7 ± 5.2% decrease (*p* < 0.001 vs. TG-treated control). At lower concentrations (1.25 and 5 μM), a dose-dependent reduction in BiFC fluorescence was observed; however, these decreases did not reach statistical significance (Figure 6C). In contrast, compounds **1**, **3**, and **4** did not exhibit notable inhibitory effects under the same conditions. To exclude the possibility that the reduced BiFC signal was attributable to cytotoxicity, cell viability was assessed in parallel by quantifying Hoechst-stained nuclei. Compound **2** showed no cytotoxicity up to 20 μM, confirming that the attenuation of tau oligomerization was not due to reduced cell survival (Figure 6D). To contextualize the biological significance of these findings, it is important to note that although the inhibitory effect of compound **2** (43.7% at 20 μM) is lower than that of classical tau aggregation inhibitors such as methylene blue, methylene blue exhibits marked cytotoxicity at comparable concentrations [39], whereas compound **2** did not affect cell viability up to 20 μM. Thus, the relevance of compound **2** should be interpreted in the context of both efficacy and safety.

Diarylheptanoids, including octahydrocurcumin analogs, are known to modulate ER stress and ROS generation—key drivers of TG-induced tau oligomerization—suggesting that compound **2** may attenuate stress-associated pathways rather than directly inhibiting tau–tau interactions. Although BiFC fluorescence is generally considered irreversible once the Venus fragments reconstitute, overall signal intensity can decline when new tau–tau interactions are suppressed or when existing complexes undergo cellular clearance. Nevertheless, because the tau-BiFC assay primarily reports early oligomerization events and cannot fully distinguish direct from indirect mechanisms, its mechanistic resolution is limited. Collectively, these considerations indicate that compound **2** confers moderate but meaningful inhibition of stress-induced tau oligomerization within a favorable cytotoxicity profile.

## 4. Conclusions

In summary, a phytochemical investigation of the EtOAc fraction of *A. agallocha* extract, guided by LC/MS-based analysis, led to the isolation and structural elucidation of four compounds: *N*-*trans*-feruloyltyramine (**1**), (3*R*,5*R*)-octahydrocurcumin (**2**), 1,7-bis(4-hydroxyphenyl)heptane (**3**), and *trans*-caffeoyltyramine (**4**). The therapeutic potential of these constituents against neurodegenerative diseases, such as Alzheimer’s disease, was assessed through evaluation of their inhibitory effects on tau protein aggregation. Among them, compound **2**, a diarylheptanoid, significantly suppressed tau oligomerization at 20 μM without exhibiting cytotoxicity in the tau–BiFC cell model. These findings highlight the potential of *A. agallocha* phytochemicals—particularly diarylheptanoids—as promising modulators of tau aggregation and underscore the pharmaceutical relevance of its secondary metabolites. This study further emphasizes the importance of exploring diverse and underinvestigated constituents of *A. agallocha* for the discovery of novel neuroprotective agents.

## Figures and Tables

**Table 1:** Equipment used for analyses.

Experimental Procedure	Equipment	
Optical rotations	JASCO P-2000 polarimeter (JASCO, Easton, MD, USA)	
Ultraviolet (UV) spectra	Agilent 8453 UV-visible spectrophotometer (Agilent Technologies, Santa Clara, CA, USA)	
Nuclear magnetic resonance (NMR) spectra	Bruker AVANCE III HD 850 NMR spectrometer (Bruker Corporation, Billerica, MA, USA) with a 5 mm TCI CryoProbe operating at 850 MHz (^1^H) and 212.5 MHz (^13^C)	
HR-ESIMS	Agilent G6545B quadrupole time-of-flight mass spectrometer (Agilent Technologies, Santa Clara, CA, USA) Agilent 1290 Infinity II high-performance liquid chromatography (HPLC) (Agilent Technologies, Santa Clara, CA, USA) instrument (Agilent Eclipse Plus C18 column (2.1 × 50 mm, 1.8 μm; flow rate: 0.3 mL/min)) (Acquity^®^ UPLC BEH C18 reverse-phase column (150 mm × 2.1 mm, 1.7 µm); flow rate: 0.3 mL/min)	
Preparative HPLC	Waters 1525 Binary HPLC pump with a Waters 996 Photodiode Array Detector (Waters Corporation, Milford, MA, USA) and a Hector C18 column (250 × 21.2 mm, 5 μm; flow rate: 5 mL/min; Rstech Corporation, Daejeon, Republic of Korea)	
Semi-preparative HPLC	Waters 1525 Binary HPLC pump with a Waters 996 Photodiode Array Detector (Waters Corporation, Milford, CT, USA) Phenomenex Luna C18 column (250 × 10 mm, 10 μm; flow rate: 2 mL/min; Phenomenex, Torrance, CA, USA)	
LC/MS analysis	Agilent 1200 Series HPLC system equipped with a diode array detector and 6130 Series ESI mass spectrometer using an analytical Kinetex C18 100 Å column (100 × 2.1 mm, 5 μm; flow rate: 0.3 mL/min; Phenomenex, Torrance, CA, USA).	
Column chromatography	Silica gel 60 (230-400 mesh; Merck, Darmstadt, Germany) Sephadex LH-20 (Pharmacia, Phenomenex, Torrance, CA, USA)	
Thin-layer chromatography (TLC)	pre-coated silica gel F254 plates and RP-C18 F254s plates (Merck, Darmstadt, Germany); spots were detected under UV light or by heating following spraying with anisaldehyde-sulfuric acid.	

## Data Availability

Data available on request from the authors.
